# Intelligent Scheduling Methodology for UAV Swarm Remote Sensing in Distributed Photovoltaic Array Maintenance

**DOI:** 10.3390/s22124467

**Published:** 2022-06-13

**Authors:** Qing An, Qiqi Hu, Ruoli Tang, Lang Rao

**Affiliations:** 1 School of Artificial Intelligence, Wuchang University of Technology, Wuhan 430223, China; 120160450@wut.edu.cn (Q.A.); 120100373@wut.edu.cn (L.R.); 2 School of Intelligent Construction, Wuchang University of Technology, Wuhan 430223, China; 120160868@wut.edu.cn; 3 School of Naval Architecture, Ocean and Energy Power Engineering, Wuhan University of Technology, Wuhan 430063, China

**Keywords:** remote sensing, unmanned aerial vehicle swarm, photovoltaic equipment maintenance, evolutionary optimization, particle swarm optimization

## Abstract

In recent years, the unmanned aerial vehicle (UAV) remote sensing technology has been widely used in the planning, design and maintenance of urban distributed photovoltaic arrays (UDPA). However, the existing studies rarely concern the UAV swarm scheduling problem when applied to remoting sensing in UDPA maintenance. In this study, a novel scheduling model and algorithm for UAV swarm remote sensing in UDPA maintenance are developed. Firstly, the UAV swarm scheduling tasks in UDPA maintenance are described as a large-scale global optimization (LSGO) problem, in which the constraints are defined as penalty functions. Secondly, an adaptive multiple variable-grouping optimization strategy including adaptive random grouping, UAV grouping and task grouping is developed. Finally, a novel evolutionary algorithm, namely cooperatively coevolving particle swarm optimization with adaptive multiple variable-grouping and context vector crossover/mutation strategies (CCPSO_-mg-cvcm_), is developed in order to effectively optimize the aforementioned UAV swarm scheduling model. The results of the case study show that the developed CCPSO_-mg-cvcm_ significantly outperforms the existing algorithms, and the UAV swarm remote sensing in large-scale UDPA maintenance can be optimally scheduled by the developed methodology.

## 1. Introduction

In recent years, the unmanned aerial vehicle (UAV) remote sensing technology has been widely used in different engineering application, e.g., soil property estimation [1], forest structure assessment [2], traffic control [3], urban infrastructure management [4], emergency scenarios [5], and so on. Due to the relatively low flying altitude, the UAV can easily acquire detailed information of observed objects with a spatial resolution under one decimeter. This advantage allows the UAV to be further applied in the maintenance of urban distributed PV arrays (UDPA), e.g., 3D reconstruction and location optimization, hot-spot detection, shading detection, cleanliness detection, and some other maintenance tasks.

With the fast development of photovoltaic (PV) power generation technology, the UDPA are widely installed in every possible corner of the city to maximize the utilization of solar energy [6,7,8]. To obtain the effective maintenance of these distributed PV arrays, the UAV remote sensing technology are widely adopted [9,10,11]. With the utilization of UAV swarm, a lot of design and maintenance tasks can be effectively accomplished, e.g., 3D reconstruction of distributed PV roofs, PV array location optimization, PV panels and infrastructures status monitoring, and so on. The application of remote sensing UAV in distributed PV infrastructures maintenance can significantly increase the work efficiency, which is of great meaningful for the optimal operation of a large-scale distributed renewable energy system.

However, due to the increasingly large number of UDPA, the efficiency requirements for UDPA maintenance tasks always cannot be satisfied by a single UAV. Instead, the UAV swarm consisting of a certain number of UAVs is adopted to accomplish the remote sensing and maintenance tasks of large-scale UDPA. Obviously, the scheduling problem for the UAV swarm significantly affects the entire maintenance efficiency. Especially, to optimally schedule the UAV swarm becomes extremely difficult when the UAV number and UDPA scale are large, as the complexity of the scheduling problem increasing exponentially with the dimensionality (i.e., the so-called “curse of dimensionality”).

Recently, worldwide scholars have become concerned with the optimization and robust control of UAV swarm or other autonomous vehicles. For example, Gu et al. concerned the problem of event-triggered secure path tracking control of autonomous ground vehicles under deception attacks, and a novel learning-based event-triggered mechanism was developed [12]. Niu et al. formulated the UAV task-scheduling problem for disaster scenarios as a two-stage Lyapunov optimization problem. They developed a dispersed computing network consisting of UAVs and ground mobile devices, which could be used for collaborative computing. Compared with the UAV-based local computation, their developed methodology could reduce the system energy consumption by more than 50% [13]. Liu et al. were concerned with the UAV swarm scheduling method for remote sensing observations in emergency scenarios. According to their experimental results, the proposed method could optimally allocate the tasks to each UAV and significantly outperforms the direct allocation method and manual scheduling method [6]. Hanna et al. proposed a method to optimize the UAV positions to maximize the MIMO capacity when a UAV swarm communicates with a distant multiantenna ground station, and their simulation results showed the robustness of their method under UAV motion disturbances [14]. Phung et al. developed a spherical vector-based particle swarm optimization algorithm to solve the path-planning problem of a UAV swarm in complicated environments [15]. As discussed above, most of the existing studies concern the scheduling, robust control or path/position optimization for a single target (e.g., a single autonomous vehicle or UAV), or for small-scale UAV swarm. However, for the UDPA system in a modern city, a lot of PV arrays with different scales are widely distributed in different city corners. When the UAV swarm is employed for the maintenance of UDPA, complexity of the scheduling problem will increase exponentially with the model dimensionality (i.e., the number of distributed PV arrays and the UAVs to be scheduled). As a result, the effective model and optimization algorithm for solving the high-dimensional UAV swarm scheduling problem are worth developing.

In this study, a novel high-dimensional scheduling model and optimization algorithm for UAV swarm remote sensing in UDPA maintenance are developed. To be specific, the UAV swarm scheduling tasks in UDPA maintenance are described as a large-scale global optimization (LSGO) problem, in which the task-allocation constraint and UAV duration constraint are introduced as penalty functions. Then, a novel adaptive multiple variable-grouping optimization strategy including the adaptive random grouping, UAV grouping and task grouping are developed. Finally, a novel evolutionary algorithm namely cooperatively coevolving particle swarm optimization with adaptive multiple variable-grouping and context vector crossover/mutation strategies (CCPSO_-mg-cvcm_) are developed to optimize the aforementioned model.

## 2. UAV Swarm Scheduling Model Based on Large-Scale Global Optimization

### 2.1. UAV Swarm Remote Sensing in UDPA Maintenance

**Figure 1:**
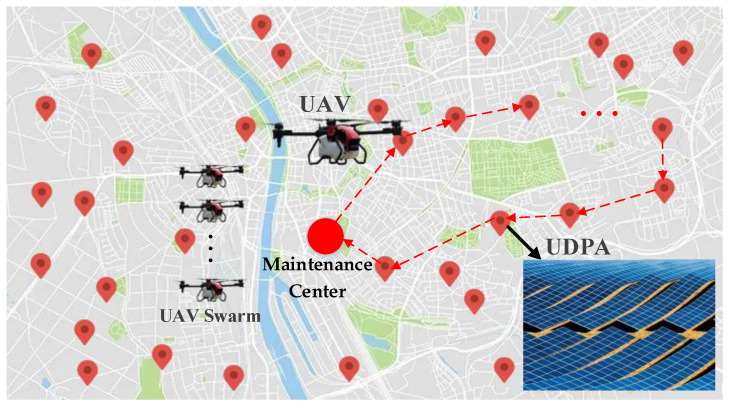
Application of UAV swarm remote sensing in UDPA maintenance.

When the UAV swarm is applied in UDPA maintenance, the UAVs should be equipped with necessary sensors, for example, the RGB camera, light-weight thermal infrared sensors, and the global positioning system/inertial measurement unit (GPS/IMU). Then, the UAVs fly to each UDPA from the maintenance center to execute the maintenance task and acquire the dataset. The application of UAV swarm remote sensing in UDPA maintenance is illustrated in Figure 1. As shown in Figure 1, for the UDPA maintenance problem, a large number of distributed PV arrays need to be maintained by the UAVs. However, the number of UAVs is significantly smaller than that of UDPAs. As a result, how to effectively schedule the UAV swarm to go to each UDPA location in turn and accomplish the detection tasks will significantly affect the overall maintenance efficiency.

**Figure 2:**
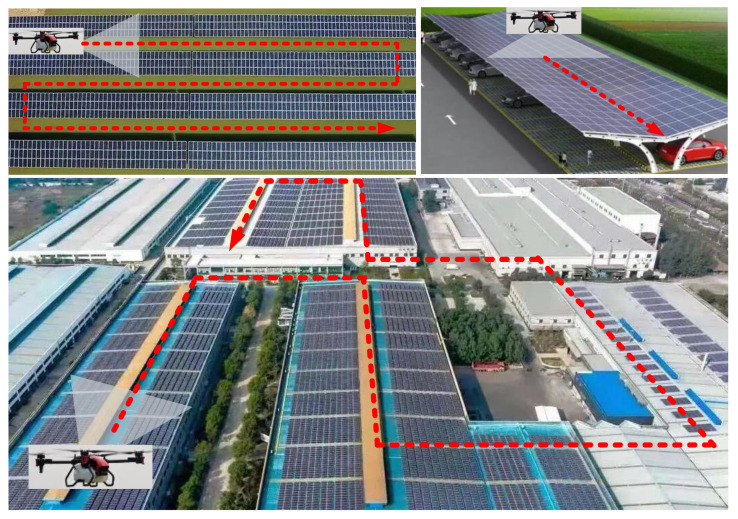
Dataset acquisition process for each UDPA.

When a UAV arrives at a certain UDPA, it needs to hover around the PV array with a certain route and scan all the PV panels to acquire the dataset. Then, when all the PV panels have been scanned, the UAV flies to the next UDPA and repeat the above work. Obviously, the time cost for the maintenance of each UDPA directly depends on its scale. The dataset acquisition process for each UDPA is illustrated in Figure 2.

Assume the UAV swarm contains *M* UAVs, and the total number of UDPA tasks is *N*. The scheduling problem is to optimally allocate the *N* tasks to the *M* UAVs and maximize the maintenance efficiency (i.e., minimize the total time cost). Denote *L_i_*_,*j*_ as the distance between the *i*th and *j*th UDPA locations, and denote *S_i_* as the hover distance when maintaining the *i*th UDPA. Then, the total time cost for the *m*th UAV, i.e., *C_m_* can be formulized as

(1) $$C_{m}=\frac{L_{0,T_{1}^{m}}+L_{T_{Nm}^{m},0}}{v_{f}}+\sum_{n=1}^{N_{m}-1}\frac{L_{T_{n}^{m},T_{\left(n+1\right)}^{m}}}{v_{f}}+\sum_{n=1}^{N_{m}}\frac{S_{T_{n}^{m}}}{v_{m}}$$

where *v_f_* is the speed of a UAV from one UDPA to another, and *v_m_* is the speed of a UAV when it hovers around the PV array and scans the PV panels to acquire the dataset. $L_{0,T_{1}^{m}}$ denotes the distance between the maintenance center and the first task location of the *m*th UAV; $L_{T_{Nm}^{m},0}$ denotes the distance between the last task location and the maintenance center; $L_{T_{n}^{m},T_{\left(n+1\right)}^{m}}$ denotes the distance between the *n*th and the (*n* + 1)th task locations of the *m*th UAV; $S_{T_{n}^{m}}$ denotes the hover distance when maintaining the $T_{n}^{m}$ th UDPA; *N_m_* is the number of tasks allocated to the *m*th UAV, and $T_{n}^{m}$ is the *n*th tasks in the task queue of the *m*th UAV.

Obviously, the entire UAV swarm scheduling problem can be formulized as

(2) $$\min\;C=\sum_{m=1}^{M}\left(\frac{L_{0,T_{1}^{m}}+L_{T_{Nm}^{m},0}}{v_{f}}+\sum_{n=1}^{N_{m}-1}\frac{L_{T_{n}^{m},T_{\left(n+1\right)}^{m}}}{v_{f}}+\sum_{n=1}^{N_{m}}\frac{S_{T_{n}^{m}}}{v_{m}}\right)$$

where *C* denotes the total time cost of a certain scheduling solution, and *M* denotes the number of UAVs in the UAV swarm.

### 2.2. Encoding and Decoding Schemes

**Figure 3:**
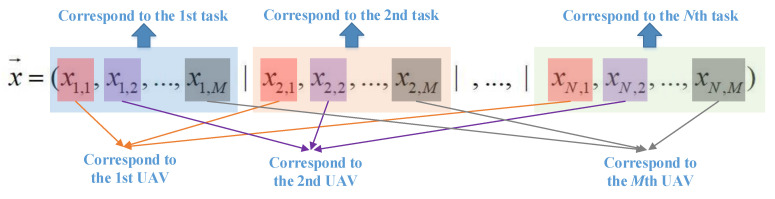
Encoding scheme for UAV swarm scheduling.

In order to minimize the total scheduling time cost using evolutionary algorithm, an efficient encoding scheme is required. In this study, for the scheduling problem with *M* UVAs and *N* task locations, N×M variables *x_n,m_* (*n* = 1, 2, …, *N*; *m* = 1, 2, …, *M*) are introduced to encode the scheduling solution. To be specific, the optimization vector $\vec{x}$, which is a combination of all the optimization variables, is defined as

(3) $$\vec{x}=(x_{1,1},\;x_{1,2},\;\ldots ,\;x_{1,M},\;\ldots ,\;x_{N,1},\;x_{N,2},\;\ldots ,\;x_{N,M})$$

where *x_n_*_,*m*_∈[0, *H_x_*] (*n* = 1, 2, …, *N*; *m* = 1, 2, …, *M*; *H_x_* represents the upper bound of *x_n,m_*, which denotes the allocating relationship between the *m*th UAV and the *n*th task. *x_n,m_* ≥ *H_x_*/2 represents the *n*th task is allocated to the *m*th UAV, and *x_n,m_* < *H_x_*/2 represents the opposite. The encoding scheme for the UAV swarm-scheduling problem is illustrated in Figure 3.

**Figure 4:**
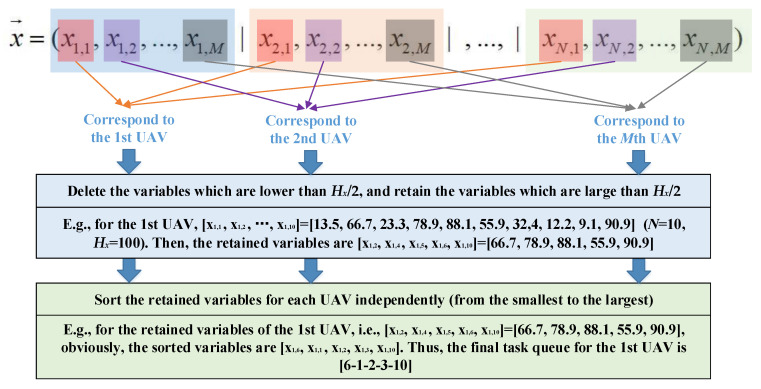
Decoding scheme for UAV swarm scheduling.

For decoding the optimization vector, the optimization variables related to a certain UAV, say the *m*th UAV, are employed to establish the its task queue. To be specific, for the *m*th UAV, the variables within *x*_1,*m*_, *x*_2,*m*_, …, *x_n_*_,*m*_ and greater than *H_x_*/2 are selected. Then, these selected variables are sorted from the smallest to the largest in order to form the final task queue of the *m*th UAV. The decoding scheme for the UAV swarm scheduling problem is illustrated in Figure 4.

### 2.3. Constraints and Penalty Function

(1) UAV duration constraint

Due to the limited flight duration of UAV, the total distance for completing the task queue of a certain UAV should be strictly constrained. To be specific, the above UAV duration constraint can be formulized as

(4) $$C_{m}=L_{0,T_{1}^{m}}+L_{T_{Nm}^{m},0}+\sum_{n=1}^{N_{m}-1}L_{T_{n}^{m},T_{\left(n+1\right)}^{m}}+\sum_{n=1}^{N_{m}}S_{T_{n}^{m}}\leq \eta_{d}\cdot L_{d-max}$$

where *C_m_* denotes the total distance for completing the task queue of a certain UAV; *L_d-max_* denotes the maximum duration for each UAV; $\eta_{d}\in (0,\;1)$ is a coefficient to ensure a certain residual power.

(2) Task-allocation constraint

Consider that in a feasible solution, each of the UDPA tasks should be allocated to a certain UAV. As a result, the task-allocation constraint can be formulized as

(5) $$\sum_{m=1}^{M}x_{n,m}^{binary}=1\;(n=1,\;2,\;\ldots ,\;N),\;x_{n,m}^{binary}=\{\begin{array}{c} 0,\;if\;x_{n,m}<\frac{H_{x}}{2}\;\\ 1,\;if\;x_{n,m}\geq \frac{H_{x}}{2} \end{array}$$

where $x_{n,m}^{binary}\in \text{\{}0,\;1\text{\}}$ denotes the binary coding variable for $x_{n,m}$.

(3) UAV-utilization constraint

In order to make full use of all the UAVs in service, the task queue for each UAV should not be empty. As a result, the UAV-utilization constraint can be formulized as

(6) $$\sum_{n=1}^{N}x_{n,m}^{binary}\geq 1\;(m=1,\;2,\;\ldots ,\;M),\;x_{n,m}^{binary}=\{\begin{array}{c} 0,\;if\;x_{n,m}<\frac{H_{x}}{2}\;\\ 1,\;if\;x_{n,m}\geq \frac{H_{x}}{2} \end{array}$$

(4) Penalty function

In order to effectively optimize the scheduling model using an evolutionary algorithm, the aforementioned constraints are defined as penalty functions and directly introduced into the optimization model. To be specific, the penalty function for a certain solution $\vec{x}$ is defined as

(7) $$C_{p}(\vec{x})=\lambda_{p}\cdot (\sum_{m=1}^{M}D_{m}+N_{task}+N_{uav}),\;D_{m}=\{\begin{array}{c} \;0,\;if\;C_{m}\leq \eta_{d}\cdot L_{d-max}\\ \frac{C_{m}}{\eta_{d}\cdot L_{d-max}},\;if\;C_{m}>\eta_{d}\cdot L_{d-max} \end{array}$$

where $C_{p}(\vec{x})$ denotes the total penalty value for a certain solution $\vec{x}$; $\lambda_{p}$ is a pre-defined coefficient to control the penalty strength; *D_m_* indicates whether the task queue for the *m*th UAV can satisfy the UAV duration constraint. *D_m_* is equal to 0 when the UAV duration constraint for the *m*th UAV is satisfied, and *D_m_* is equal to $C_{m}/\eta_{d}\cdot T_{d-max}>1$ when the constraint is broken; *N_task_*∈[0, N] denotes the number of tasks that do not satisfy the task-allocation constraint; *N_uav_*∈[0, M] denotes the number of UAVs that do not satisfy the UAV-utilization constraint. Obviously, for a feasible solution, *D_m_* (*m* = 1, 2, …, *M*), *N_task_* and *N_uav_* should be equal to 0.

As discussed above, the overall UAV swarm scheduling model by considering all the constraints can be formulized as

(8) $$\min\;f=C+C_{p}=\sum_{m=1}^{M}\left(\frac{L_{0,T_{1}^{m}}+L_{T_{Nm}^{m},0}}{v_{f}}+\sum_{n=1}^{N_{m}-1}\frac{L_{T_{n}^{m},T_{\left(n+1\right)}^{m}}}{v_{f}}+\sum_{n=1}^{N_{m}}\frac{S_{T_{n}^{m}}}{v_{m}}\right)+\lambda_{p}\cdot (\sum_{m=1}^{M}D_{m}+N_{task}+N_{uav})$$

## 3. A Novel CCPSO_-mg-cvcm_ Optimization Algorithm

As discussed in Section 2, the optimal scheduling for remote sensing a UAV swarm is modeled as a N×M dimensional optimization problem. Obviously, the model complexity significantly increases with the number of UAVs and the scale of the UDPA system. For example, when 20 UAVs (*M* = 20) are employed to execute the maintenance tasks of 80 UDPAs (*N* = 80), the model dimensionality is 20×8=1600. Obviously, this ultra-high dimensional problem is too complex to be optimized by using traditional optimization algorithms. In order to overcome this problem, a novel evolutionary algorithm, namely, the cooperatively coevolving particle swarm optimization with adaptive multiple variable-grouping and context vector crossover/mutation strategies (CCPSO_-mg-cvcm_), is developed.

### 3.1. Particle Swarm Optimization

With the fast development of artificial intelligence (AI) theories and technologies, many AI-based algorithms are developed and employed for solving real-world problems, for example, in remote sensing [16,17], renewable energy system [18], architecture [19,20], human behavior recognition [21], control of unmanned vehicles [22] and so on [23]. The evolutionary algorithm (EA) is an important branch of AI technology, and it is widely employed for solving complex optimization problems [24]. In recent years, many EA-based methodologies are widely developed and obtain promising performance in solving real-world optimization problems [25,26,27].

Particle swarm optimization (PSO) is an evolutionary algorithm typically employed in numerical optimization problems. The idea of PSO originates from the imitation of foraging behavior of swarms such as birds and fishes. When PSO is employed to optimize a certain problem, all the individuals (also called the particle) in its population are randomly initialized, and each presents a potential solution for the original problem. Then, according to the velocity and position updating formulas, each particle searches for the optimal position in the solution space by iteratively updating. When the stopping criteria are reached, the algorithm outputs the best particle in the newest generated population as the final solution. The basic evolution rules in PSO can be formulized as

(9) $$v_{i}(t+1)=\omega \cdot v_{i}(t)+\alpha_{l}\cdot (x_{i-best}(t)-x_{i}(t))+\alpha_{g}\cdot (x_{gbest}(t)-x_{i}(t))$$

(10) $$x_{i}(t+1)=x_{i}(t)+v_{i}(t+1)$$

where *x_i_*(*t*) and *v_i_*(*t*) denote the position and velocity of the *i*th particle in the *t*th generation; *x_i-best_*(*t*) denotes the historical best position for the *i*th particle in the *t*th generation; *x_gbest_*(*t*) denotes the global best position in the *t*th generation; $\alpha_{l}$ and $\alpha_{g}$ are coefficients to control the cognitive and social learning strength. In recent years, many PSO variants have been developed and employed to solve different engineering problems [28].

### 3.2. Cooperatively Coevolving

In the optimization of high-dimensional problem, the basic PSO and most of its variants always lose their efficacies due to the “curse of dimensionality”. In order to overcome this problem, the cooperatively coevolving (CC) inspired by the “divide and conquer” philosophy is developed [29]. In the CC framework, the original *D*-dimensional problem is decomposed into several relatively low-dimensional sub-problems. Then, all the sub-problems are coevolved one-by-one in each generation. As each sub-problem only contains a part of variables of the original problem, it is impossible to compute the fitness function value without a context. As a result, one or more *D*-dimensional particles are defined as the context vector (CV) to provide references for the variables corresponding to the other dimensionalities.

For the UAV scheduling model described in Section 2, the application of the PSO and CC framework is illustrated in the following steps:

**Step 1**: For the original D=N×M dimensional scheduling model (denoted as *UAV-Model*_0_), initialize the PSO population with *N_P_* particles. Each particle represents a *D*-dimensional optimization vector as listed in Equation (3). Then, decompose the original *UAV-Model*_0_ into *K* sub-models (denote as *Sub-Model_i_*, *i* = 1, 2, …, *K*), i.e., *UAV-Model*_0_ = (*Sub-Model*_1_, *Sub-Model*_2_, *…*, *Sub-Model_K_*). Note that the dimensionality of each sub-model is equal to *D/K.* Then, each *D*-dimensional particle (i.e., optimization vector) $\vec{x}$ can be denoted as $\vec{x}$ = (*x*^1^, *x*^2^, …, *x^K^*), where *x^i^* represents the corresponding variables (i.e., dimensionalities) belongs to the *i*th sub-model, i.e., *Sub-Model_i_*.

**Step 2**: Denote the selected context vector as ***CV***. Then, the *Sub-model_i_* in the CC framework can be defined as

(11) $$\min\;f(x^{i}|CV),\;x^{i}\in {\left[0,\;H_{x}\right]}^{D/K},\;CV\in {\left[0,\;H_{x}\right]}^{D}$$

where $f(x^{i}|CV)=f(CV^{1},\;\ldots ,\;CV^{i-1},\;x^{i},\;CV^{i+1},\;\ldots ,\;CV^{K})$ represents the fitness function for *Sub-model_i_*.

**Step 3**: Start an evolution cycle. All the sub-models as listed in Equation (11) are optimized one-by-one using PSO.

**Step 4:** Proceed another evolution cycle if the stopping criteria are not satisfied; otherwise, stop the cooperatively coevolving process and output the *D*-dimensional global best particle.

### 3.3. Adaptive Multiple Variable-Grouping Strategy

Actually, in the CC framework as discussed in Section 3.2, the decomposition strategy of the original model significantly affects the optimization performance. That is to say, how to allocate the original *D* variables to each sub-model is very important. In order to ensure that the heavily coupled variables are grouped into the same sub-model and coevolved for enough iterations, a novel multiple variable-grouping strategy is developed. To be specific, all the optimization variables *x_n,m_* (*n* = 1, 2, …, *N*; *m* = 1, 2, …, *M*) are grouped using the following strategies:

(1) Adaptive random grouping

The most widely used variable-grouping strategy is random grouping, which means that all the variables are randomly disordered and grouped into each sub-model. In this study, the adaptive random grouping (AR-grouping) is developed, in which the group size can be adaptively adjusted. Steps for the AR-grouping strategy are as follows:

**Step 1:** Randomly disorder the entire *D* dimensionalities within the original model.

**Step 2:** Decompose the disordered dimensionalities into K = *D*/*s* sub-models, where *s* is a pre-defined variable to control the size of each sub-model. Randomly increase or decrease *s* with a certain step-size Δs, i.e., set s=s±Δs.

**Step 3:** In each iteration, check whether the global optimum is effectively evolved. Keep increasing (or decreasing) *s* with the same direction if the global optimum is evolved; otherwise, change the disturbance direction of the group-size *s*. Then, go to Step 1 to re-disorder and re-decompose the dimensionalities with the newly updated *s*.

**Figure 5:**
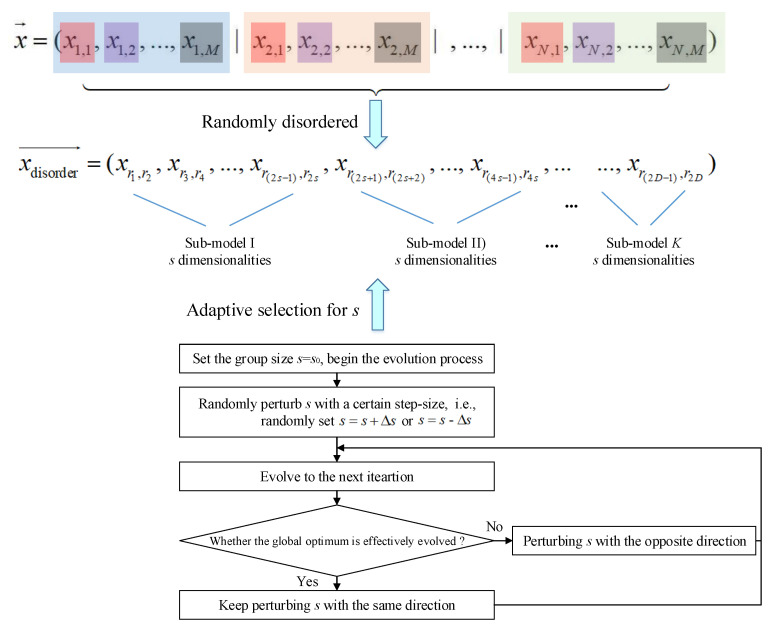
Schematic for AR-grouping strategy.

The schematic of the AR-grouping strategy is illustrated in Figure 5.

(2) UAV variable grouping

In the UAV variable grouping strategy (denote as UAV-grouping), the variables related to a certain UAV are grouped into the same sub-model. To be specific, set the group size *s* to k·N(*k* = 1, 2, …, *M*). When *s* = *N* in a certain iteration, the variables for the first UAV (denote as UAV_1_), i.e., *x*_1,1_, *x*_2,1_, …, *x_n_*_,1_, are regarded as the first sub-model; then, the variables for the second UAV (denote as UAV_2_), i.e., *x*_1,2_, *x*_2,2_, …, *x_n_*_,2_, are regarded as the second sub-model, and so on. Similarly, when *s* = k·N in a certain iteration, the variables for the adjacent *k* UAVs are grouped into the same sub-model. That is to say, *x*_1,1_, …, *x_n_*_,1_, *x*_1,2_, …, *x_n_*_,2_, …, *x*_1,*k*_, …, *x_n,k_*, are regarded as the first sub-model; then, *x*_1,(*k* + 1)_, …, *x_n_*_,(*k* + 1)_, *x*_1,(*k* + 2)_, …, *x_n_*_,(*k* + 2)_, …, *x*_1,2*k*_, …, *x_n_*_,*2k*_, are regarded as the second sub-model, and so on.

**Figure 6:**
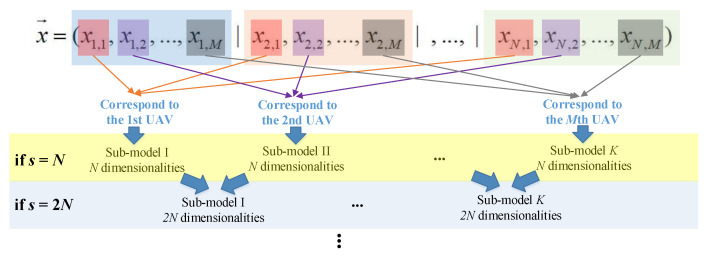
Schematic for UAV-grouping strategy.

The schematic of the UAV-grouping strategy is illustrated in Figure 6.

(3) Task variable grouping

In the Task variable grouping strategy (denote as Task-grouping), the variables related to a certain UDPA are grouped into the same sub-model. Similar with the UAV-grouping, set the group size s to k·M(*k* = 1, 2, …, *N*). When *s* = *M* in a certain iteration, the variables for the first UDPA (denote as UDPA_1_), i.e., *x*_1,1_, *x*_1,2_, …, *x*_1,*m*_, are regarded as the first sub-model; then, the variables for the second UDPA (denote as UDPA_2_), i.e., *x*_2,1_, *x*_2,2_, …, *x*_2,*m*_, are regarded as the second sub-model, and so on. Similarly, when s =k·M in a certain iteration, the variables for the adjacent *k* UDPAs are grouped into the same sub-model. That is to say, *x*_1,1_, …, *x*_1,*m*_, *x*_2,1_, …, *x*_2,*m*_, …, *x_k_*_,1_, …, *x_k,m_* are regarded as the first sub-model; then, *x*_(*k*_ _+_ _1),1_, …, *x*_(*k* + 1),*m*_, *x*_(*k* + 2),1_, …, *x*_(*k* + 2),*m*_, …, *x*_2*k*,1_, …, *x*_2*k*,*m*_, are regarded as the second sub-model, and so on.

**Figure 7:**
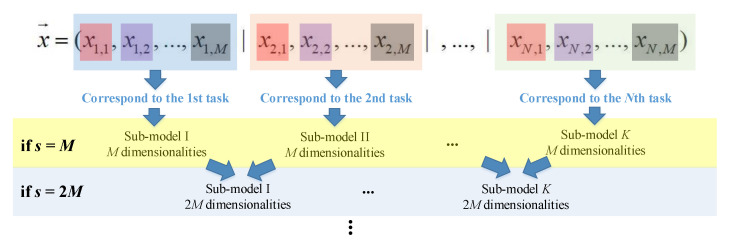
Schematic for Task-grouping strategy.

The schematic of the Task-grouping strategy is illustrated in Figure 7.

In the overall adaptive multiple variable-grouping strategy, the AR-grouping, UAV-grouping and Task-grouping are adaptively selected in each iteration. To be specific, each of the grouping strategies are randomly selected according to their probabilities, which are adaptively updated in each iteration. The adaptive probabilities for each grouping strategy are defined as

(12) $$\{\begin{array}{c} \rho_{\mathrm{AR}}=\frac{N_{AR}}{N_{AR}+N_{UAV}+N_{TASK}}\\ \rho_{UAV}=\frac{N_{UAV}}{N_{AR}+N_{UAV}+N_{TASK}}\\ \rho_{TASK}=\frac{N_{TASK}}{N_{AR}+N_{UAV}+N_{TASK}} \end{array}$$

where $\rho_{AR}$, $\rho_{UAV}$ and $\rho_{TASK}$ represent the adaptive probabilities for AR grouping, UAV grouping and Task grouping, respectively; *N_AR_*, *N_UAV_* and *N_TASK_* represent the probability coefficients for each grouping strategy. The principles for updating these probability coefficients are as follows:

Firstly, *N_AR_*, *N_UAV_* and *N_TASK_* are all initialized to *N*_0_ (e.g., *N*_0_ = 5) at the beginning of iteration. That is to say, the AR-grouping, UAV-grouping and Task-grouping strategies have the same probabilities (i.e., 33.33% for each one) to be selected at the beginning;

Then, check whether the global optimum is evolved at the end of each iteration. Add 1 to *N_AR_* (or *N_UAV_*, or *N_TASK_*) if the AR-grouping (or UAV-grouping, or Task-grouping) strategy is selected in this iteration and the global optimum is effectively evolved.

**Figure 8:**
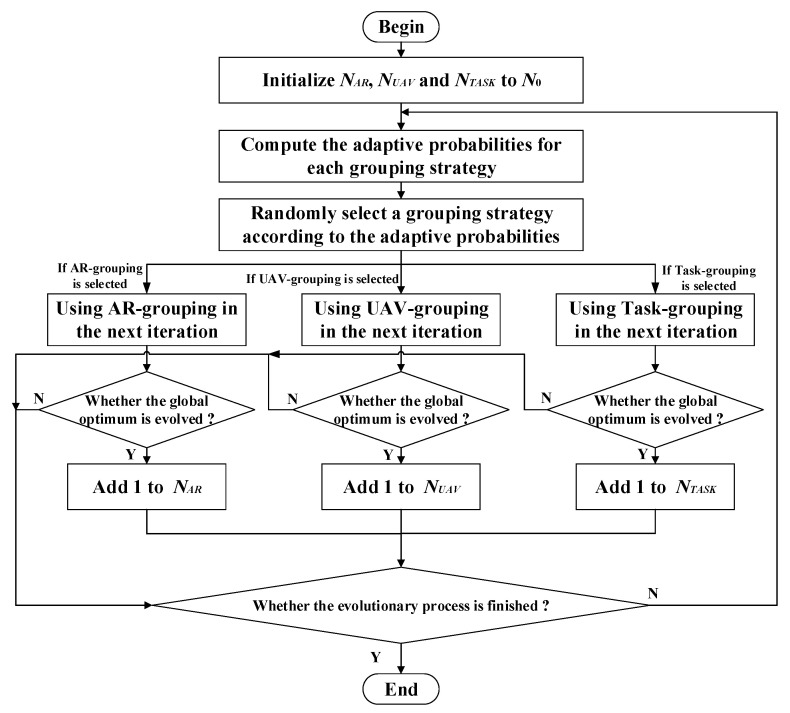
Flowchart of adaptive multiple variable-grouping strategy.

As discussed above, the flowchart of the adaptive multiple variable-grouping strategy is illustrated in Figure 8.

### 3.4. Context Vector Crossover and Mutation Strategy

As discussed in Section 3.2, when the CC framework is employed to solve a high-dimensional optimization problem, one or more *D*-dimensional vectors should be defined as the context vector to provide references when evolving a certain part of the original problem. In this study, the best (*p_cv_* − 1) particles in the current population and another randomly generated particle in the current population are employed as the context vector. Note that *p_cv_* denotes the total number of CV.

(1) CV crossover strategy

**Figure 9:**
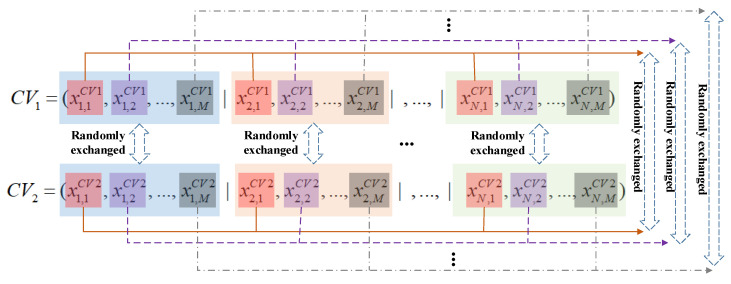
Schematic for CV crossover strategy.

In order to keep the diversity of CV, the crossover mechanism is introduced. To be specific, randomly select two CVs, say CV_1_ and CV_2_, and randomly exchange part of the dimensionalities in CV_1_ and CV_2_. Note that in the CV crossover strategy, the exchanged dimensionalities are always corresponding to the same UAV or the same UDPA. After the crossover operation, update the original CVs (i.e., CV_1_ and CV_2_) using the newly generated CVs (denote as CV_1-new_ and CV_2-new_) if better. The schematic of the CV crossover strategy is illustrated in Figure 9.

(2) CV mutation strategy

As discussed in Section 2.3, each of the UDPA tasks should be allocated to a certain UAV. That is to say, in a feasible scheduling solution, each of the variable groups related to a certain UDPA should be a one-hot vector, i.e., only one variable in *x_n_*_,1_, *x_n_*_,2_, …, *x_n_*_,*M*_ (*n* = 1, 2, …, *N*) is greater than *H_x_*/2. As a result, the CV mutation strategy is developed in the following steps:

**Step 1:** Define the parameter $\rho_{m}\in [0,\;1]$ to control the mutation probabilities for each CV;

**Step 2:** For each of the *p_cv_* CVs, say the *i*th CV*_i_*, randomly generate a mutation variable $\rho_{i}$ within the interval [0, 1]. Then, mutate CV*_i_* according to the following principles:

- If $\rho_{i}\leq \rho_{m}$, keep CV*_i_* unchanged;
- Otherwise, each of the components [*x_n_*_,1_, *x_n_*_,2_, …, *x_n_*_,*M*_] (*n* = 1, 2, …, *N*) in CV*_i_* is randomly mutated to [*r*_0_, *r*_0_, *…*, *r*_0_, *r*_1_], [*r*_0_, *…*, *r*_0_, *r*_1_, *r*_0_], …, [*r*_0_, *r*_1_, *r*_0_, *…*, *r*_0_] and [*r*_1_, *r*_0_, *r*_0_, …, *r*_0_], in which each *r*_0_ is randomly generated within [0, *H_x_*/2) and each *r*_1_ is randomly generated within [*H_x_*/2, *H_x_*].

**Step 3:** Denote the mutated CV as *CV_i_*_-*mut*_. Update *CV_i_* using *CV_i_*_-*mut*_ if better.

### 3.5. The Overall CCPSO_-mg-cvcm_ Optimization Algorithm

By integrating the aforementioned CC framework, adaptive multiple variable-grouping strategy and the CV crossover/mutation strategy, a novel evolutionary algorithm namely CCPSO_-mg-cvcm_ is developed to optimize the high-dimensional UAV swarm scheduling model. In the CCPSO_-mg-cvcm_, the position updating principle for each particle follows the AMCCPSO developed in our previous study [30]. The developed CCPSO_-mg-cvcm_ algorithm is illustrated in Algorithm 1.
**Algorithm 1.** Pseudo code of CCPSO_-mg-cvcm_.Initialize D=N×M dimensional *Population* with *N_P_* particles. Initialize *p_cv_* context vectors with the best (*p_cv_* − 1) particles and a randomly selected particle. **repeat**  Update the adaptive probabilities for each grouping strategy, and randomly select a grouping strategy according to these probabilities.  Decompose the original *D*-dimensional model into several sub-models using the selected grouping strategy. Denote the *j*th sub-model as *Sub-Model_j_*.     Execute the CV crossover operation for *N_mu_* times. Execute the mutation operation for each CV.   **for** *each Sub-Model_j_* **do**
    Coevolve the corresponding variables using AMCCPSO principles [30].   **end****until** the stopping criteria are satisfied.

### 3.6. Application of the Overall UAV Swarm Scheduling Methodology

In the real-world application, the scheduling model described in Section 2 is established by using the real problem details: for example, the number of tasks and UAVs, the detailed locations and scales of each task, the distance duration and speed for each UAV and so on. Then, run the CCPSO_-mg-cvcm_ algorithm and output the global best solution. Finally, decode the best solution and obtain the task sequence for each UAV. As discussed above, the aforementioned scheduling model and optimization algorithm are implemented in the following steps:

**Step 1**: Collect the model parameters according to the real scheduling problem, including the number of tasks and UAVs, the locations and scales of each task, the maximum distance duration and speed of UAV;

**Step 2:** Establish the LSGO-based high-dimensional scheduling model by using the collected parameters in Step 1;

**Step 3**: Run the CCPSO_-mg-cvcm_ algorithm and output the global best solution;

**Step 4:** Decode the best solution output in Step 3 and obtain the task sequence for each UAV;

**Step 5**: Transmit the task sequence obtained in Step 4 to each UAV.

## 4. Case Studies and Analysis

### 4.1. Experimental Setup

**Figure 10:**
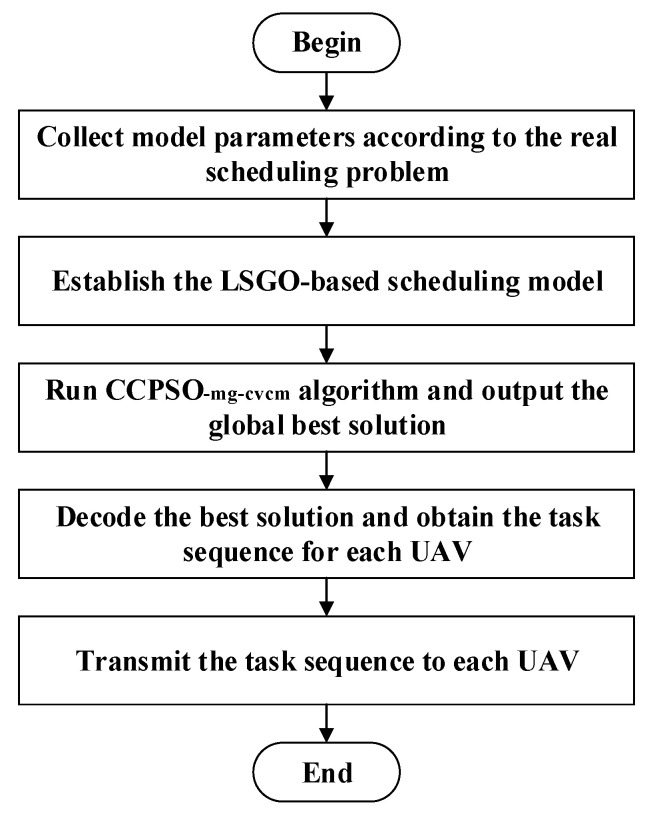
Flowchart of the developed UAV swarm scheduling methodology in practical application.

In order to verify the effectiveness of the developed UAV swarm scheduling model and optimization algorithm, some numerical experiments for different cases are conducted in this section. Assume the UAV swarm contains 10 remote sensing UAVs, and there are in total 50 UDPAs that need to be maintained. The location and scale for each UDPA are shown in Figure 10. Note that in Figure 10, the red circle represents the maintenance center, i.e., the beginning and ending points for the UAV swarm. The small blue circles denote the 50 UDPAs located in different areas of a city, and the number next to each point denotes the scale for each UDPA (i.e., the hover distance of the UAV when maintaining the corresponding UDPA). The X and Y axes represent the horizontal and vertical distances between the UDPA and maintenance center, and the units are kilometers. The detailed parameter settings employed in the following experiments are listed in Table 1.

### 4.2. Experimental Results and Analysis

**Figure 11:**
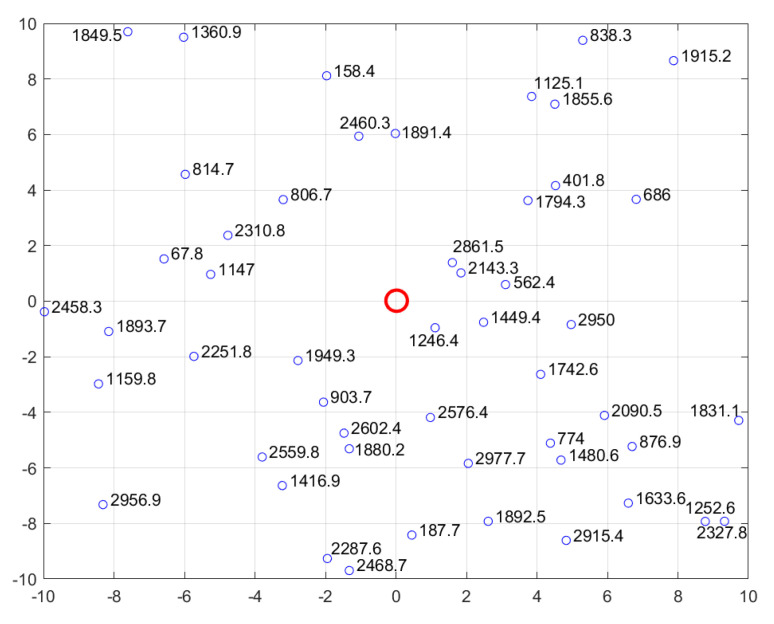
Location and scale for 50 UDPAs.

**Figure 12:**
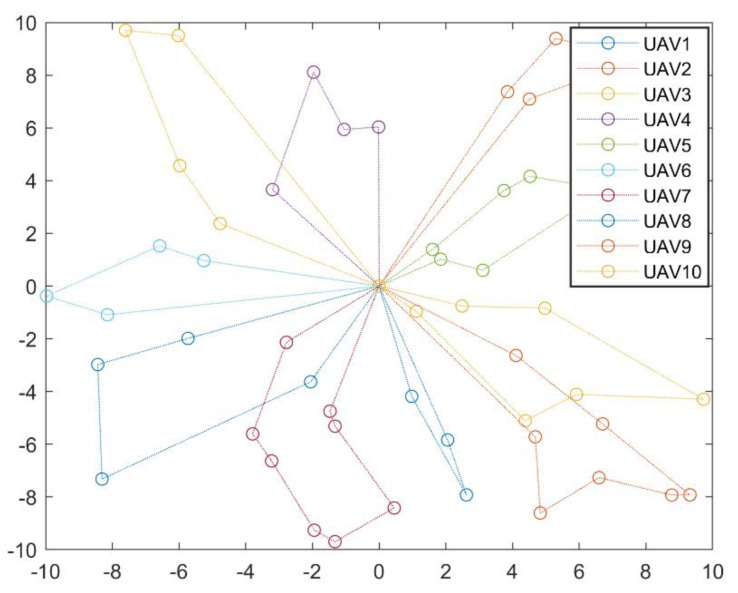
Optimal scheduling solution for the UAV swarm obtained by CCPSO_-mg-cvcm_.

For the UAV swarm scheduling problem as shown in Figure 11, the optimal scheduling solution obtained by CCPSO_-mg-cvcm_ is listed in Table 2 and plotted in Figure 12. The final optimized fitness function value is 40.45, which is smaller than the pre-defined penalty strength coefficient $\lambda_{p}$ = 10,000. This implies that all the constraints are strictly satisfied in the optimized scheduling solution.

**Figure 13:**
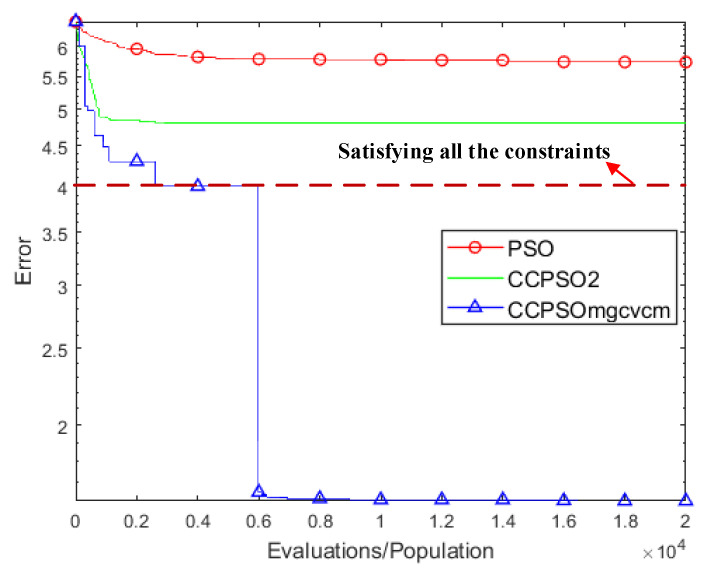
Convergence graphs for the compared algorithms.

In order to verify the effectiveness of the CC framework, multiple variables-grouping strategy, CV crossover and mutation strategies, the basic PSO and the famous CCPSO2 algorithm are employed for comparison [29]. The final fitness function values obtained by each algorithm are compared in Table 3. As shown in the table, the performance of CCPSO_-mg-cvcm_ significantly outperforms its competitors. To be specific, for the basic PSO and the CCPSO2 algorithms, the final fitness function values are all significantly larger than the pre-defined penalty strength coefficient $\lambda_{p}$. This implies that due to the high dimensionality, some of the constraints are broken and the model is punished by the defined penalty functions. However, the final fitness function value obtained by CCPSO_-mg-cvcm_ is just 40.45, which is significantly less than the penalty strength coefficient $\lambda_{p}=$ 10,000. This implies that all the model constraints are satisfied in the final solution output by CCPSO_-mg-cvcm_, and the total cost is effectively optimized. The convergence graphs for PSO, CCPSO2 and CCPSO_-mg-cvcm_ are compared in Figure 13.

### 4.3. Analysis for Different Model Dimensionalities

**Figure 14:**
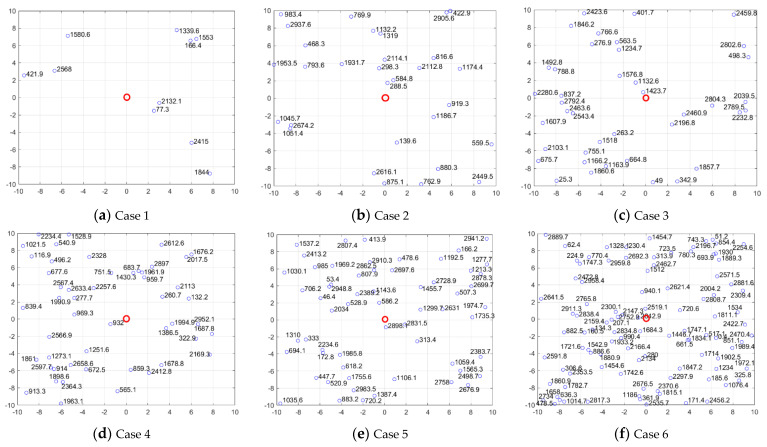
Location and scale of UDPAs for each case.

**Figure 15:**
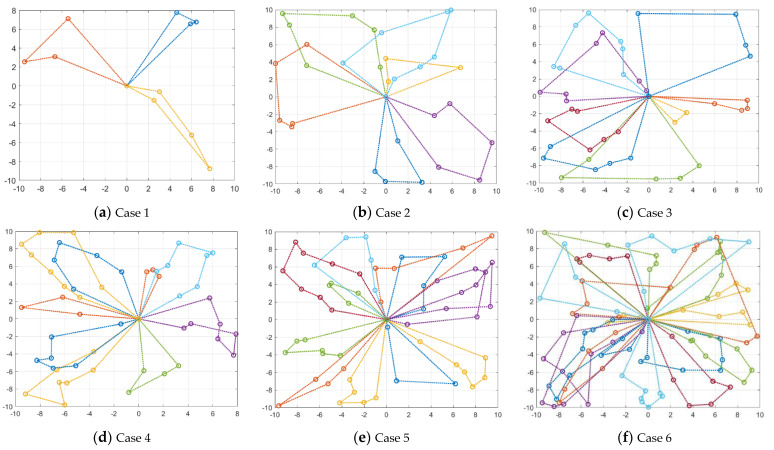
Optimal scheduling solution for each case.

In order to further analyze the performance of CCPSO_-mg-cvcm_ for different model dimensionalities (i.e., different scales for UAV swarm and UDPA system), some more case studies are conducted in this section. The detailed parameter settings for each case are listed in Table 4. The locations and scales for UDPAs in each case are plotted in Figure 14. The final scheduling solutions obtained by CCPSO_-mg-cvcm_ for each case are shown in Figure 15. Note that in Figure 15, each line represents the flight path of a single UAV. 

As shown in Figure 15, the developed CCPSO_-mg-cvcm_ performs robustly for all the cases, and it can effectively output the optimal scheduling solution of the UAV swarm. Especially for Case 6, in which the model dimensionality increases to 2400, the developed CCPSO_-mg-cvcm_ can effectively optimize such a high-dimensional problem and also satisfy all the constraints. The detailed task queues for each UAV in Case 6 are listed in Table 5. As shown in the table, due to the different scale and location of each UDPA, the lengths of UAV task queues are different from each other. For example, the task queue lengths for UAV_1_, UAV_5_, UAV_6_, UAV_15_ and UAV_19_ are 7; however, the lengths for UAV_3_, UAV_8_ and UAV_10_ are only 3. Note that the task queue for UAV_11_ is empty, which implies that the overall 120 UDPAs can be maintained by 19 UAVs with low time-cost and can satisfy all the constraints, so UAV_11_ is excluded from the UAV swarm.

### 4.4. Comparison for Different Algorithms

In order to further verify the outperformance of CCPSO_-mg-cvcm_ in optimizing a high-dimensional scheduling model, some state-of-the-art evolutionary algorithms are employed for comparison. The compared algorithms include the basic CPSO-SK_-rg-aw_ [31], CCPSO2 [29], JADE [32] and AMCCDE [33]. The population sizes of all of the compared algorithms are set to 50, and the maximum number of fitness evaluations is set to 2 × 10^6^. The other parameter settings of the compared algorithms are the same as their original studies. Parameter settings for Case 7–10 are listed in Table 6. As shown in Table 6, the model dimensionality significantly increases from Case 7 to Case 10. To be specific, in Case 7, the numbers of UAV and UDPA are 6 and 30, respectively, and the model dimensionality is equal to 6 × 30 = 180. In Case 8, the numbers of UAV and UDPA increase to 12 and 60, respectively, and the model dimensionality increases to 12 × 60 = 720. Finally, the model dimensionalities in Case 9 and 10 further increase to 1200 and 2000. Obviously, the high-dimensionality characteristics in Cases 9 and 10 will significantly increase the problem complexity and lead to the failure of some traditional optimization algorithms.

The results of simulations for different algorithms are compared in Table 7. As shown in the table, the developed CCPSO_-mg-cvcm_ obtains the best performance for all the cases. For example, when optimizing the 180-dimensional model in Case 7, CCPSO2, AMCCDE and CCPSO_-mg-cvcm_ can satisfy all the constraints, because the final results obtained by these algorithms are significantly less than the penalty strength coefficient $\lambda_{p}=$ 10,000. Specifically, the performance obtained by CCPSO_-mg-cvcm_ for Case 7 (i.e., 25.251) is better than CCPSO2 (29.142) and AMCCDE (27.599). This implies that the scheduling solution provided by the developed CCPSO_-mg-cvcm_ has a higher efficiency than that of CCPSO2 and AMCCDE, and the overall time-cost for accomplishing all the maintenance tasks is significantly reduced by CCPSO_-mg-cvcm_. However, for the compared CPSO-SK_-rg-aw_ and JADE algorithms, some constraints are broken in the final solution, so the final cost values obtained by CPSO-SK_-rg-aw_ and JADE are significantly larger than the other algorithms.

For the 720-dimensional model in Case 8, the final results obtained by CPSO-SK_-ra-aw_, CCPSO2, JADE and AMCCDE are all significantly larger than the penalty strength coefficient $\lambda_{p}$. This indicates that the scheduling solutions obtained by these algorithms are infeasible because some of the constraints are not satisfied. However, the final result obtained by CCPSO_-mg-cvcm_ is just 43.747, which is significantly smaller than the penalty strength coefficient $\lambda_{p}$. Obviously, this implies that the CCPSO_-mg-cvcm_ can satisfy all the constraints and output efficient scheduling solution for this 720-dimensional problem.

When the dimensionality further increases to 1200 in Case 9 and 2000 in Case 10, all the compared algorithms lose their efficacies and cannot efficiently optimize the high-dimensional model. However, the developed CCPSO_-mg-cvcm_ can obtain robust performance, the final cost value obtained by CCPSO_-mg-cvcm_ is 61.996 in Case 9 and 80.628 in Case 10. It can be concluded that the developed CCPSO_-mg-cvcm_ algorithm can obtain promising performance on optimizing the high-dimensional UAV swarm scheduling model with up to more than 2000 dimensionalities.

## 5. Conclusions

This study concerns the unmanned aerial vehicle (UAV) swarm scheduling problem when applied to remote sensing in urban distributed photovoltaic arrays (UDPA) maintenance. On one hand, the UAV swarm scheduling model and the penalty function-based constraints are established. On the other hand, a novel evolutionary algorithm, namely cooperatively coevolving particle swarm optimization with adaptive multiple variable-grouping and context vector crossover/mutation strategies (CCPSO_-mg-cvcm_), is developed to optimize the scheduling model. The results of case study show that the dimensionality of the scheduling model significantly increases with the scales of UAV swarm and UDPA to be maintained, and most of the existing algorithms lose their efficacies when adopted to optimize these high-dimensional problems. However, with the integration of a cooperatively coevolving framework, adaptive multiple variable-grouping strategy, and context vector crossover/mutation strategies, the developed CCPSO_-mg-cvcm_ significantly outperforms the existing algorithms, and it can effectively optimize the high-dimensional (even up to 2400 dimensionalities) scheduling model with robust performance. In the future, the deep learning-based technique will be examined to further improve the performance of UAV swarm remote sensing under a complex environment [34].

## Figures and Tables

**Table 1:** Detailed parameter settings for the scheduling model and optimization algorithm.

Parameter	Meaning	Value	Parameter	Meaning	Value
*v_f_*	Speed of UAV from one UDPA to another	25 m/s	*v_m_*	Speed of UAV when hover around PV array	15 m/s
*L_d-max_*	Maximum distance duration for each UAV	40 km	$\eta_{d}$	Power consumption coefficient for UAV	90%
*H_x_*	Upper bound of optimization variables	100	$\lambda_{p}$	Penalty strength coefficient	10,000
Δs	Step-size for random grouping	5	*s_0_*	Initial group size for random grouping	10
*p_cv_*	Number of CV	5	*N_0_*	Initial value for probability coefficients	5
*N_p_*	Number of particle in the PSO swarm	50	$\rho_{m}$	Mutation probability control coefficient	0.7
*Max_ges*	Maximum evaluation number for CCPSO_-mg-cvcm_	2 × 10^6^	*N_mu_*	Number of CV crossover operation	5

**Table 2:** Optimal task queues for each UAV obtained by CCPSO_-mg-cvcm_.

UAV	Task Queue	UAV	Task Queue
UAV_1_	8----20----41----39	UAV_6_	11----40----23----13
UAV_2_	35----25----47----37	UAV_7_	5----48----45----14----6----44----33----10
UAV_3_	31----7----24----21----15----26	UAV_8_	50----2----1
UAV_4_	38----30----34----27	UAV_9_	49----29----4----3----42----43----9
UAV_5_	19----16----12----32----36----22	UAV_10_	17----28----18----46

**Table 3:** Comparison for the final fitness function value of PSO, CCPSO2 and CCPSO_-mg-cvcm_.

Algorithm	Final Fitness Function Value	Algorithm	Final Fitness Function Value	Algorithm	Final Fitness Function Value
PSO	547,478.43	CCPSO2	64,563.43	CCPSO_-mg-cvcm_	40.45

**Table 4:** Parameter settings for each case.

Cases	Number of UAV	Number of UDPA	Model Dimensionality	Maximum Evaluation Number
Case 1	3	10	30	2 × 10^5^
Case 2	6	30	180	5 × 10^5^
Case 3	8	40	320	1 × 10^6^
Case 4	10	50	500	1 × 10^6^
Case 5	14	80	1120	1 × 10^6^
Case 6	20	120	2400	1 × 10^6^

**Table 5:** Optimal task queues for each UAV in Case 6.

UAV	Task Queue	UAV	Task Queue
UAV_1_	25----27----40----50----58----93----95	UAV_11_	/
UAV_2_	43----44----74----80----89	UAV_12_	13----47----49----69----70
UAV_3_	18----36----45	UAV_13_	23----53----76----81
UAV_4_	8----29----39----78----83----87	UAV_14_	6----9----10----32----48----68
UAV_5_	7----24----30----37----61----94----98	UAV_15_	2----15----51----77----85----91----99
UAV_6_	31----34----35----46----66----73----96	UAV_16_	5----19----52----57----88
UAV_7_	16----33----38----72----79	UAV_17_	11----26----28----63
UAV_8_	1----14----67	UAV_18_	22----54----55----75----90
UAV_9_	59----62----64----86----100	UAV_19_	3----17----20----41----71----82----84
UAV_10_	12----21----56	UAV_20_	4----42----60----65----92----97

**Table 6:** Parameter settings for Case 7 to Case 10.

Cases	Number of UAV	Number of UDPA	Model Dimensionality	Maximum Evaluation Number
Case 7	6	30	180	1 × 10^6^
Case 8	12	60	720	1 × 10^6^
Case 9	15	80	1200	1 × 10^6^
Case 10	20	100	2000	1 × 10^6^

**Table 7:** Comparison for different optimization algorithms.

Cases	CPSO-SK_-rg-aw_	CCPSO2	JADE	AMCCDE	CCPSO_-mg-cvcm_
Case 7	7.2602 × 10^4^	**2.9142 × 10^1^**	1.6330 × 10^4^	**2.7599 × 10^1^**	**2.5251 × 10^1^**
Case 8	1.3087 × 10^5^	6.8420 × 10^4^	7.4197 × 10^5^	3.3308 × 10^4^	**4.3747 × 10^1^**
Case 9	2.3357 × 10^5^	1.0856 × 10^5^	1.8142 × 10^6^	8.4918 × 10^4^	**6.1996 × 10^1^**
Case 10	3.3947 × 10^5^	1.3128 × 10^5^	4.4930 × 10^6^	9.4073 × 10^4^	**8.0628 × 10^1^**

Note that the solutions satisfied all the constraints are set in bold.

## References

[1] Ivushkin K., Bartholomeus H., Bregt A.K., Pulatov A., Franceschini M.H.D., Kramer H., van Loo E.N., Jaramillo Roman V., Finkers R. (2019). UAV based soil salinity assessment of cropland. Geoderma.

[2] Wallace L., Lucieer A., Malenovský Z., Turner D., Vopěnka P. (2016). Assessment of forest structure using two UAV techniques: A comparison of airborne laser scanning and structure from motion (SfM) point clouds. Forests.

[3] Zhu J.S., Sun K., Jia S., Li Q.Q., Hou X.X., Lin W.D., Liu B.Z., Qiu G.P. (2018). Urban Traffic Density Estimation Based on Ultrahigh-Resolution UAV Video and Deep Neural Network. IEEE J. Sel. Top. Appl. Earth Obs. Remote Sens..

[4] Congress S.S.C., Puppala A.J., Lundberg C.L. (2018). Total system error analysis of UAV-CRP technology for monitoring transportation infrastructure assets. Eng. Geol..

[5] Liu J.L., Liao X.H., Ye H.P., Yue H.Y., Wang Y., Tan X., Wang D.L. (2022). UAV swarm scheduling method for remote sensing observations during emergency scenarios. Remote Sens..

[6] Choi Y., Rayl J., Tammineedi C., Brownson J.R.S. (2011). PV Analyst: Coupling ArcGIS with TRNSYS to assess distributed photovoltaic potential in urban areas. Sol. Energy.

[7] Liao W., Xu S., Heo Y. (2022). Evaluation of model fidelity for solar analysis in the context of distributed PV integration at urban scale. Build. Simul..

[8] Shafique M., Luo X.W., Zuo J. (2020). Photovoltaic-green roofs: A review of benefits, limitations, and trends. Solar Energy.

[9] Hwang Y.S., Schluter S., Park S.I., Um J.S. (2021). Comparative evaluation of mapping accuracy between UAV video versus photo mosaic for the scattered urban photovoltaic panel. Remote Sens..

[10] Niccolai A., Grimaccia F., Leva S. (2019). Advanced asset management tools in photovoltaic plant monitoring: UAV-based digital mapping. Energies.

[11] Li X.X., Yang Q., Chen Z.B., Luo X.J., Yan W.J. (2017). Visible defects detection based on UAV-based inspection in large-scale photovoltaic systems. IET Renew. Power Gener..

[12] Gu Z., Yin T.T., Ding Z.T. (2021). Path tracking control of autonomous vehicles subject to deception attacks via a learning-based event-triggered mechanism. IEEE Trans. Neural Netw. Learn. Syst..

[13] Niu Z.C., Liu H., Lin X.M., Du J.Z. (2022). Task scheduling with UAV-assisted dispersed computing for disaster scenario. IEEE Syst. J..

[14] Hanna S., Krijestorac E., Cabric D. (2021). UAV swarm position optimization for high capacity MIMO backhaul. IEEE J. Sel. Areas Commun..

[15] Phung M.D., Ha Q.P. (2021). Safety-enhanced UAV path planning with spherical vector-based particle swarm optimization. Appl. Soft Comput..

[16] Wang S.F., Zhou J., Zhong H.L. (2020). Estimating land surface temperature from satellite passive microwave observations with the traditional neural network, deep belief network, and convolutional neural network. Remote Sens..

[17] Chang Y.L., Tan T.H., Lee W.H., Chang L.A., Chen Y.N., Fan K.C., Alkhaleefah M. (2022). Consolidated convolutional neural network for hyperspectral image classification. Remote Sens..

[18] Tang R.L., Wu Z., Li X. (2018). Optimal operation of photovoltaic/battery/diesel/cold-ironing hybrid energy system for maritime application. Energy.

[19] An Q., Chen X., Wang H., Yang H., Yang Y. (2022). Segmentation of concrete cracks by using fractal dimension and UHK-net. Fractal Fract..

[20] An Q., Chen X.J., Zhang J.Q., Shi R.Z., Yang Y.J., Huang W. (2022). A robust fire detection model via convolution neural networks for intelligent robot vision sensing. Sensors.

[21] Liu H., Nie H., Zhang Z., Li Y.-F. (2021). Anisotropic angle distribution learning for head pose estimation and attention understanding in hu-man-computer interaction. Neurocomputing.

[22] Gu Z., Ahn C.K., Yan S., Xie X.P., Yue D. (2022). Event-triggered filter design based on average measurement output for networked unmanned surface vehicles. IEEE Trans. Circuits Syst. II Express Briefs.

[23] Liu H., Zheng C., Li D., Zhang Z., Lin K., Shen X., Xiong N.N., Wang J. (2022). Multi-perspective social recommendation method with graph representation learning. Neurocomputing.

[24] Shaikh P.W., El-Abd M., Khanafer M., Gao K.Z. (2022). A review on swarm intelligence and evolutionary algorithms for solving the traffic signal control problem. IEEE Trans. Intell. Transp. Syst..

[25] Li X.T., Ma S.J., Hu J.H. (2017). Multi-search differential evolution algorithm. Appl. Intell..

[26] Zhong X.X., Cheng P. (2021). An elite-guided hierarchical differential evolution algorithm. Appl. Intell..

[27] Chen X., Tianfield H., Du W.L. (2021). Bee-foraging learning particle swarm optimization. Appl. Soft Comput..

[28] Tang R.L., Li X., Lai J.G. (2018). A novel optimal energy-management strategy for a maritime hybrid energy system based on large-scale global optimization. Appl. Energy.

[29] Li X.D., Yao X. (2012). Cooperatively coevolving particle swarms for large-scale optimization. IEEE Trans. Evol. Comput..

[30] Tang R.L., Wu Z., Fang Y.J. (2017). Adaptive multi-context cooperatively coevolving particle swarm optimization for large-scale problems. Soft Comput..

[31] Li X.D., Yao X. Tackling high dimensional nonseparable optimization problems by cooperatively coevolving particle swarms. Proceedings of the 2009 IEEE Congress on Evolutionary Computation.

[32] Zhang J.Q., Sanderson A.C. (2009). JADE: Adaptive differential evolution with optional external archive. IEEE Trans. Evol. Comput..

[33] Tang R.L., Li X. (2018). Adaptive multi-context cooperatively coevolving in differential evolution. Appl. Intell..

[34] Liu H., Liu T., Zhang Z., Sangaiah A.K., Yang B., Li Y.F. (2022). ARHPE: Asymmetric Relation-aware Representation Learning for Head Pose Estimation in Industrial Hu-man-computer Interaction. IEEE Trans. Ind. Inf..

